# Supplementary material for: Efficient Toughening of Short-Fiber Composites Using Weak Magnetic Fields
Source: Materials (Basel). 2020 May 25;13(10):2415. doi: 10.3390/ma13102415 (PMC7288324; doi:10.3390/ma13102415)
Supplement: Supplementary file 1 [file materials-13-02415-s001.pdf]

materials

Supplementary Information

Efficient Toughening of Short Fiber Composites Using Weak Magnetic Fields

Omri Goldberg, Israel Greenfeld and Hanoch Daniel Wagner

S1.Curing System

Left: A photograph of the concentration/alignment combined setup (setup 3)

Right: A photograph of the alignment setup, a home-made solenoid which supplies uniform and unidirectional magnetic flux (setup 2)

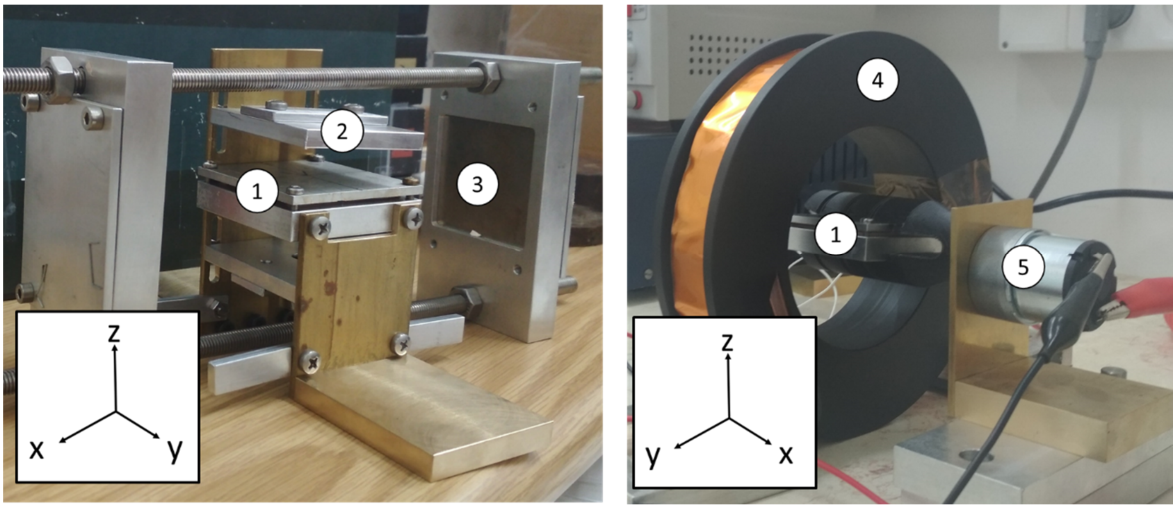

Figure S1. Photographs of the curing setups. 1—Pre-cured compact-tension sample sealed in an aluminum container, 2—Concentration Magnets H_c, 3—Bias Magnets H_b, 4—Solenoid, 5—Rotation Motor.

S2. Photographic Analysis

Contour maps of compact tension samples cured by means of various magnetic configurations with different ratios of H_c/H_b.

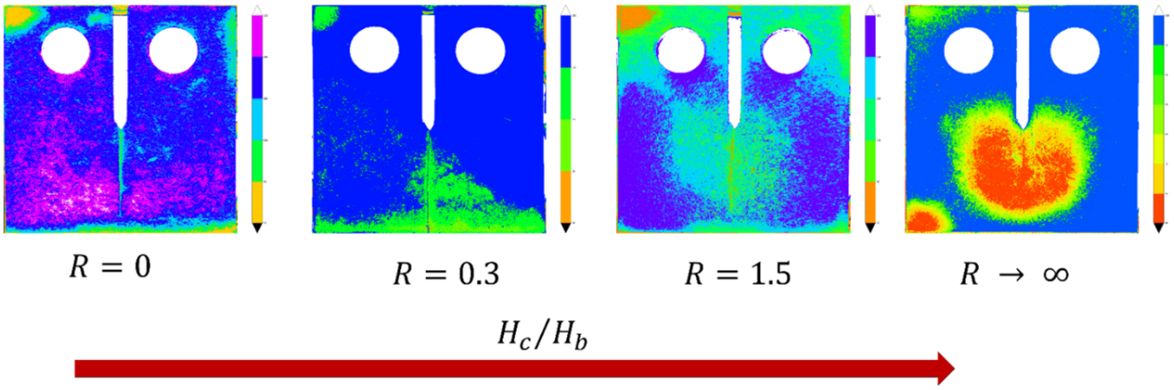

Figure S2. Contour maps of compact-tension samples

Rough estimation of the densest configuration (setup 1, $R = \frac{H_c}{H_b} \to \infty$):

Total area of the composite = 136 − 16 = 120 (arb. unit)

Concentrated Area = 34 (arb. unit)
The upper limit for the concentration efficiency factor in the current system is thus:

$$\eta_c \approx \frac{120}{34} \approx 4 \tag{S1}$$

S3. Fracture-Toughness Results-Instron

EP-502/EPC-9, control: 0.86 ± 0.21 [MPa·m$^{0.5}$]

Table S1. Fracture toughness measurements of composites with different fiber coating.

	GFus	GFs	GFus-MAG	GFs-MAG
2 wt.%	0.86 ± 0.30	0.81 ± 0.34	0.76 ± 0.16	0.91 ± 0.33
10 wt.%	0.83 ± 0.20	0.98 ± 0.10	1.00 ± 0.08	0.99 ± 0.12
20 wt.%	1.20 ± 0.15	1.14 ± 0.09	1.35 ± 0.12	1.28 ± 0.09

S4. Model

Using the general relationship between the fracture toughness (K_{IC}), the elastic modulus(E) and the toughness (G_c) a general model was developed to examine the effect of fiber orientation and concentration. Note: The calculation is not a prediction, only a description of the behavior of different composites and their relationships with the volume fraction and the orientation.

The parameters specified in the model are specified in the following Table S2:

Table S2. Model parameters.

Symbol	Description	Units	Notes
C	Crack geometry factor	-	Remains constant
$G_{c,m}$	toughness of the matrix	J/m^2	Matrix surface energy per unit area
$G_{c,f}$	toughness of the fiber	J/m^2	or G_i, fiber-matrix surface energy per interfacial unit area
${\eta_\theta}^E$	Krenchel factor	-	orientation of modulus
${\eta_l}^E$	Fiber lengths factor	-	According to Cox model ~0.25
${\eta_c}^E$	Modulus concentration factor	-	Designated in previous paper[1] as χ_d where ${V_f}^{eff} = V_f\chi_d$
${\eta_\theta}^G$	Toughening orientation factor	-	Calculated as[2] ${\eta_\theta}^G = cos(\theta)exp\left(\frac{\mu}{2}\theta\right)$
${\eta_l}^G$	Geometrical factor to translate fiber embedded area to composite cross-section area	-	l_f/d_f, fiber aspect ratio
${\eta_c}^G$	Toughening density factor	-	Analogous to modulus
μ	Snubbing Friction Coefficient	Rad^{-1}	<0.3

Starting with these three basic relationships:

$(a)\ K_{IC} = C\sqrt{EG_c}$

$(b)\ E = \left(1 - {\eta_c}^E V_f\right)E_m + {\eta_\theta}^E {\eta_l}^E {\eta_c}^E V_f E_f$

$(c)\ G_c = \left(1 - {\eta_c}^G V_f\right)G_{c,m} + {\eta_\theta}^G {\eta_l}^G {\eta_c}^G V_f G_{c,f}$

the following expression is obtained,

$$\frac{K_{IC}}{K_{IC,0}} = \sqrt{\frac{G_c \cdot E}{G_{c,m} \cdot E_m}} = \sqrt{{\eta_\theta}^E {\eta_l}^E {\eta_c}^E V_f \frac{E_f}{E_m} + \left(1 - {\eta_c}^E V_f\right)}\sqrt{{\eta_\theta}^G {\eta_l}^G {\eta_c}^G V_f \frac{G_{c,f}}{G_{c,m}} + \left(1 - {\eta_c}^G V_f\right)}, \tag{S2}$$

The expression for $\frac{K_{IC}}{K_{IC,0}}$ contains three components: The contribution of the fibers, the contribution of the matrix and a cross-coupling factor.

$$\frac{K_{IC}}{K_{IC,0}} = \sqrt{\begin{array}{c}\left[(\eta_\theta{}^E\eta_l{}^E\eta_c{}^E)(\eta_\theta{}^G\eta_l{}^G\eta_c{}^G)\frac{E_f}{E_m}\frac{G_{c,f}}{G_{c,m}}\right]V_f{}^2 + \\ \left[\eta_\theta{}^E\eta_l{}^E\eta_c{}^E\frac{E_f}{E_m}(1-\eta_c{}^G V_f) + \eta_\theta{}^G\eta_l{}^G\eta_c{}^G\frac{G_{c,f}}{G_{c,m}}(1-\eta_c{}^E V_f)\right]V_f + \\ (1-\eta_c{}^E V_f)(1-\eta_c{}^G V_f)\end{array}} \quad (S3)$$

Focusing on the contribution of the fibers only, neglecting the matrix contribution (leaving only the first term), and reorganizing the equation we get:

$$\left[\frac{K_{IC}}{K_{IC,0}}\right]_{\text{fibers}} = \eta_\theta{}^K\eta_c{}^K\eta_l{}^K V_f\sqrt{\frac{E_f}{E_m}\frac{G_{c,f}}{G_{c,m}}} \quad (S4)$$

where the fracture toughness efficiency factors (η^K, given in the text simply as η) are defined as geometric averages of the efficiency factors of the modulus and the toughness ($\eta^E\eta^G$):

$$\eta_c{}^K = \sqrt{\eta_c{}^E\eta_c{}^G} \cong \eta_c{}^E = \eta_c{}^G$$

$$\eta_\theta{}^K = \sqrt{\eta_\theta{}^E\eta_\theta{}^G} = cos^{2.5}(\theta) \times exp\left(\frac{\mu}{2}\theta\right) \cong cos^{2.5}(\theta) \quad (S5)$$

$$\eta_l{}^K = \sqrt{\eta_l{}^E\eta_l{}^G} \cong \sqrt{\eta_l{}^E l_f/d_f} \cong 1.85$$

Finally, by transforming the weight fraction of the fillers to volume fraction,

$$V_f = \frac{m_f\rho_m}{m_f\rho_m + m_m\rho_f}\ ,\ V_f(10\ \text{wt}\ \%) = 0.05 \quad (S6)$$

the entire expression $\frac{K_{IC}}{K_{IC,0}}$ can be calculated for our system as a function of η_c and η_θ.

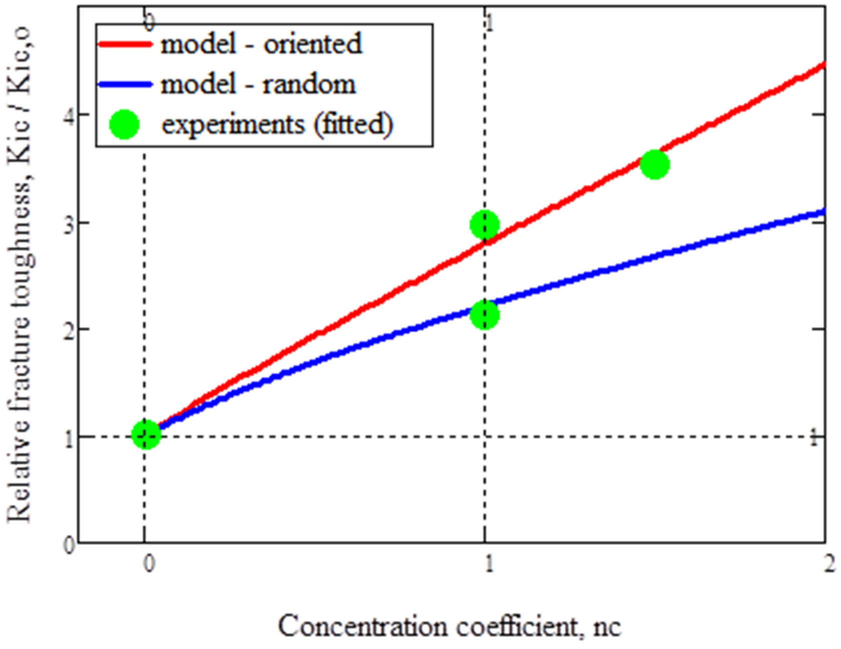

Figure S3. Comparison of modeled predictions and experimental results.

S5. The Effect of Snubbing at Inclined Angles on Pull out Energy

Calculation of the pullout energy, normalized by the pullout energy for aligned fibers ($\theta = 0^{\circ}$), as a function of the fiber orientation:

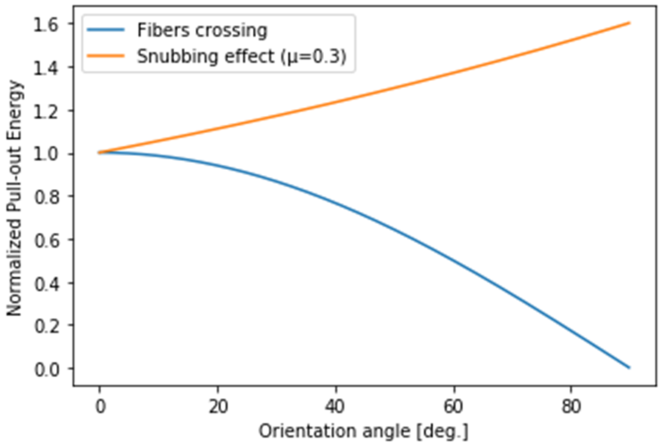

Figure S4. Effect of orientation angle on the pullout energy components

Total normalized pullout energy as a function of the snubbing coefficient μ (for epoxy μ ≈ 0.3)

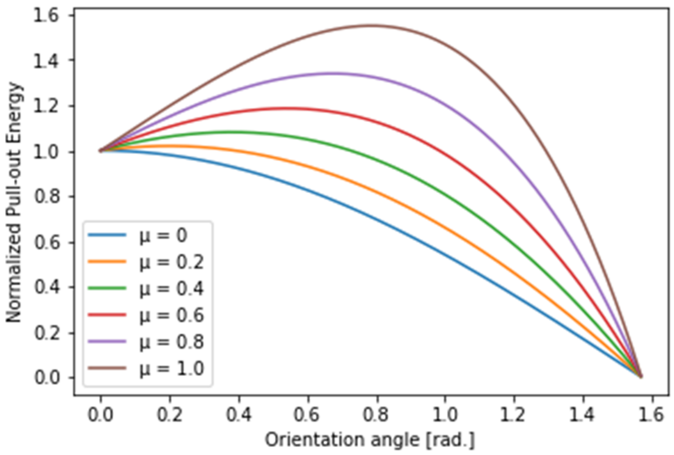

Figure S5. Effect of snubbing coefficient on the pullout energy

1. Goldberg O.; Greenfeld I.; Wagner H.D. Composite Reinforcement by Magnetic Control of Fiber Density and Orientation. *ACS Appl. Mater. Interfaces* **2018**, *10*, 16802–16811.
2. Wetherhold R.C.; Jain L.K. The Effect of Crack Orientation on the Fracture Properties of Composite Materials. *Mater. Sci. Eng. A* **1993**, *165*, 91–97.

© 2020 by the authors. Submitted for possible open access publication under the terms and conditions of the Creative Commons Attribution (CC BY) license (http://creativecommons.org/licenses/by/4.0/).
